# The overlooked impact of background diet and adherence in nutrition trials

**DOI:** 10.1101/2025.02.26.25322933

**Published:** 2025-02-26

**Authors:** Javier I. Ottaviani, Hagen Schroeter, Dennis M. Bier, John W. Erdman, Howard D. Sesso, JoAnn E. Manson, Gunter G. C. Kuhnle

**Affiliations:** 1) Mars, Inc., McLean, VA;; 2) Department of Pediatrics, Baylor College of Medicine, Houston, TX, USA; 3) Department of Food Science and Human Nutrition, University of Illinois at Urbana-Champaign, Urbana-Champaign, IL, USA.; 4) Division of Preventive Medicine, Brigham and Women’s Hospital, Harvard Medical School.; 5) Department of Food and Nutritional Sciences, University of Reading, Reading, UK;

**Keywords:** Randomized Clinical Trials, Nutrition, Biomarkers, Flavanols

## Abstract

**Importance:**

Randomized controlled trials in nutrition (RCTN) face unique challenges such as the influence of participants’ background diets and varying adherence to the intervention, factors that are difficult to quantify and can mask true intervention effects. However, the exact impact of these factors remains unclear.

**Objective:**

To quantify the impact of background diet and adherence, estimated using validated nutritional biomarkers of flavanol intake, on the outcomes of a large-scale RCTN.

**Design, Setting, and Participants:**

This study was nested within the COcoa Supplement and Multivitamin Outcomes Study (COSMOS; NCT02422745), a randomized, double-blind, placebo-controlled, 2×2 factorial trial conducted among 21,442 older adults in the United States. Analyses focused on participants (n=6,532) in the placebo and cocoa-extract intervention arms who had available biospecimens and outcome data.

**Exposures:**

Daily supplementation with a cocoa flavanol (CF).

**Main Outcomes and Measures:**

We used validated flavanol biomarkers in urine to assess background dietary flavanol intake and adherence to the cocoa extract intervention. Outcomes included total cardiovascular disease (CVD) events, CVD mortality, all-cause mortality, and major CVD events. Hazard ratios (HRs) with 95% CIs were estimated using intention-to-treat, per-protocol, and biomarker-based analyses.

**Results:**

Of the 6,532 participants, 20% in both the placebo and intervention arms had a background flavanol intake similar to that provided by the intervention; only 5% had no background flavanol intake. In the intervention group, 33% of participants were not adherent based on expected biomarker levels, that was larger than the15% estimated based on self-reported pill-taking. Accounting for biomarker-estimated background diet and adherence resulted in a significant impact on effect sizes. For total CVD events, the HR (95% CI) of 0.79 (0.59–1.05) in the per-protocol analysis (PP), was reduced to 0.65 (0.47–0.89) in the biomarker-based analysis. Similar changes were observed for CVD mortality (from 0.51 (0.23; 1.14) to 0.44 (0.20; 0.97)), all-cause mortality (0.69 (0.45; 1.05) to 0.54 (0.37; 0.80)) and major CVD events (from 0.62 (0.43; 0.91) to 0.48 (0.31; 0.74)). Overall, biomarker-based analyses consistently produced stronger effect estimates than ITT and PP analyses that do not consider the impact of background diet or assess adherence using self-reported methods.

**Conclusions and Relevance:**

These results highlight the importance of considering diet and adherence in RCTN using nutritional biomarker-based analyses. This may help to improve consistency and reliability of outcomes of RTC testing nutritional interventions.

## Introduction

Randomized controlled trials (RCTs) are generally considered the gold standard for the scientific study of drugs and treatments – both in medical (1–4) and nutrition research (5–7). However, nutrition trials (RCTN) face unique challenges (8–10) that are often overlooked. In particular, the impact of background diet of participants on trial outcomes and the objective assessment of adherence. Unlike in pharmacological RCTs where the uncontrolled and unknown exposure to the medical drug under investigation can almost never occur, participants in RCTN will almost always be exposed to foods, nutrients, or dietary constituents that are similar to or indistinguishable from the study intervention (8). Very often this exposure from background diet cannot be quantified by the investigators for integration in analyses (11). The same applies to adherence, which can be more easily monitored with pharmacological interventions where specific and unambiguous biomarkers exist (12–14), whereas most RCTN have to rely exclusively on self-reported information (15–20) – approaches that carry a higher risk of misclassification of adherence. Taken together, these two challenges can significantly affect the outcomes and mask differences between intervention and control groups, leading to incorrect interpretations. Considering the cost, efforts, and high impact of, especially large-scale RCTN, it is important to quantify the potential error introduced by these two often overlooked factors, and to identify and use better methods to address these limitations. This will allow to generate RCTN outcomes of higher scientific rigor and evidence quality, more comparable to RCT in medical research, and contribute to increase reliability and confidence in nutrition research by providing clearer messages to practitioners and the general public

Validated nutritional biomarkers provide the opportunity to address both challenges (21, 22). They measure the systemic presence of the dietary compound under investigation and thereby can provide objective information on both, background diet and adherence to the intervention. However, because there are so few validated nutritional biomarkers that have been identified (22), integration in large-scale RCTN has been rare and the actual impact is currently unknown.

Flavanols are a group of food bioactives for which a beneficial effect on health has been established (23). They have been investigated recently in a large RCT, COSMOS (COcoa Supplement and Multivitamin Outcomes Study), which showed that flavanol intake reduces CVD risk (24). The COSMOS trial included the collection of spot urine samples at baseline and during follow-up, allowing objective, biomarker-based assessments of both participants’ background diets and adherence to the intervention using validated nutritional biomarkers (23, 24). Hence, flavanols and COSMOS represent an excellent model to assess the impact that dietary background and adherence could have on the outcomes of a large RCTN.

## Results

### COSMOS Biomarker cohort

A total of 6,509 out of the 21,442 participants randomized in COSMOS provided spot urine samples for the quantification of flavanol biomarkers (gVLM_B_ and SREM_B_; see Figure S1 and Table S1 for more details).

### Background diet and adherence assessment using flavanol biomarkers

**Figure 1.**
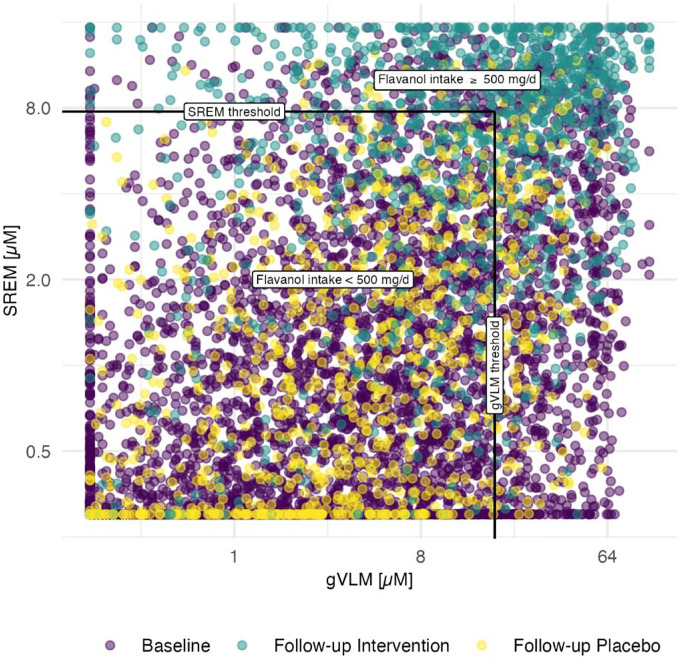
Concentration of SREM_B_ and gVLM_B_ in participants (n=6,509) at baseline and follow-up in intervention and placebo groups. Black line represents thresholds for SREM_B_ and gVLMB. Participants having gVLM_B_ or SREM_B_ levels above the corresponding thresholds were considered to have a flavanol intake equal or higher than 500 mg/d.

Biomarker concentrations at baseline and follow up in the COSMOS Biomarker cohort are shown in Figure 1. At baseline, biomarker concentration in approximately 20% of participants were consistent with flavanol intake of at least 500 mg/d as part of their background diet, with little differences between participants on the intervention and control group (Table 1). Only 5% of participants had biomarker concentrations below the limit of quantification of the analytical method, and thus, a negligible or very low exposure to flavanols from the background diet.

Among 2,051 participants with follow-up spot urine samples, 67% of those assigned to the intervention group had a flavanol consumption consistent with the intake of at least 500 mg/d of flavanols (Table 1) and were thus considered adherent to study pill intake. This figure was lower than the adherence determined using self-reported methods, which was 85% (25).

### Biomarker-based intervention groups

We used flavanol biomarker data to identify participants as either part of the *biomarker active* or *biomarker control* groups, analogous to the intervention-based *intervention* and *control* group. *Biomarker active* participants had a biomarker estimated flavanol intake of at least 500 mg/d either at baseline or follow-up. Those in the *biomarker control* group did not achieve these biomarker concentrations at baseline and at follow up. Participants without a follow up sample in the CF intervention group who had a background diet providing less than 500 mg/d of flavanols were excluded from the analysis (n=1,774; 27% of Biomarker Cohort) as adherence could not be determined in this group.

Only 62% of participants in the *biomarker active* group had been randomized into the intervention group, whereas 10% of participants in the *biomarker control* group had been randomized into the intervention. In addition to differences in the number of participants receiving placebo and intervention in the biomarker active and biomarker control groups, additional differences in the baseline characteristics of these participants are shown in Table S2.

### CVD events in biomarker-based intervention groups

Table 2 shows the associations between intervention groups and disease risk among those in the COSMOS Biomarker cohort. There were substantial differences between HRs when using biomarker-based groups and the randomized groups. The *biomarker active* group showed statistically significant reduced risks for all endpoints, while the associations were weaker when using groups based on randomization assignment. In the intention-to-treat (ITT) analysis, which compares intervention and placebo and does not take adherence and background diet into consideration, a significant reduction in risk was found only for CVD mortality, consistent with the main COSMOS trial findings (25). Taking self-reported adherence into consideration (per protocol analysis, PP), we also found significant risk reductions for major CVD events and all-cause mortality, but not total CVD events.

**Figure 2.**
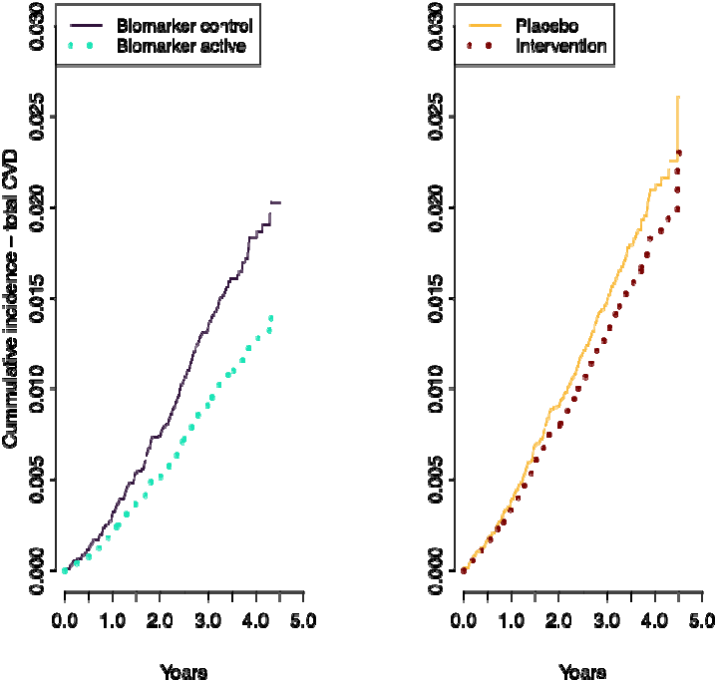
Cumulative incidence of total cardiovascular events according to biomarker-based groups (A) and randomized assignment in per-protocol analysis (B). Results shown are adjusted for age at randomization (75), sex (male), BMI (25 kg/m^2^), smoking status (never), aspirin use (no) and recruitment cohort (WHI). Total CVD events was a composite of myocardial infarction, stroke, CVD death, coronary artery bypass graft and percutaneous coronary intervention, unstable angina including hospitalization, carotid artery surgery, and peripheral artery surgery.

The cumulative incidence curves for total CVD events in the COSMOS trial and in the COSMOS biomarker cohort when using groups based on randomization showed a divergence in hazard rates from approximately one year of follow-up (Figure 2B). In contrast, when using *biomarker-based* intervention groups, divergence starts earlier (Figure 2A). This difference could be the consequence of baseline dietary flavanols mediating an effect in those participants that already had biomarker levels consistent with an intake of flavanol equal or above 500 mg/d before commencement of COSMOS trial.

### Sensitivity analyses

A series of sensitivity analyses were conducted to determine robustness of the findings for biomarker-based intervention groups (Tables S3 and S4). These analyses showed that adjusting for diet quality did not alter the results obtained, suggesting that flavanol intake, rather than a broader beneficial dietary pattern, may be responsible for the differences in CVD events between biomarker-based groups. Additional adjustment by randomization group attenuated the estimated association (from 0.65 (0.47;0.89) to 0.70 (0.48;1.02) for total cardiovascular events; from 0.44 (0.20;0;0.97) to 0.64 (0.26;1.54) for cardiovascular mortality; from 0.54 (0.37;0.80) to 0.65 (0.41;1.02) for all-cause mortality; and from 0.48 (0.31;0.74) to 0.55 (0.33; 0.91) for major cardiovascular events), which is not surprising as most participants in the *biomarker active* group are also in the CF intervention group.

## Discussion

In this study, we investigated two main limitations of RCTs in nutrition: background diet and adherence, and how these limitations could affect outcomes in such studies. For that, we used nutritional biomarkers to objectively assessed background diet and adherence, and using flavanols, flavanols biomarkers and the COSMOS trial (25) as a model system. (26), (27). Our results show that both background diet and adherence have a substantial impact on the results of RCTNs:

1. One in five participants in both the placebo and intervention arms were already consuming flavanols as part of their background diet in amounts that were similar or higher to those tested in the intervention arm, and only one in twenty participants were not consuming any flavanols at baseline.
2. Approximately one third of participants assigned to the intervention did not attain the flavanol levels as set forth in this study for this intervention.
3. Considering both biomarker-estimated dietary background and adherence to study intervention resulted in larger effect sizes than the traditional approach which neither considered background intake nor biomarker-estimated adherence.

The use of biomarkers allows the objective assessment of intake as part of the background diet and adherence to the intervention. This approach offers significant benefits compared to methods based on dietary and adherence questionnaires and food content databases, which are not only subjective in nature but known to incur significant levels of uncertainty (28, 29). In contrast to biomarkers that reflect the systemic presence, self-reported data does not capture food and nutrient interactions (30, 31) and the effects of food processing (32) that can affect systemic exposure.

When using nutritional biomarkers, it is important to consider interindividual differences in absorption, distribution, metabolism, and excretion (ADME) as a potential limitation that may lead to the misclassification of participants in the biomarker-based active and biomarker-based control groups. In the case of flavanols as used here, the impact of ADME has been previously considered during the validation of gVLM_B_ and SREM_B_ as biomarkers (23, 24, 27). In addition, the combination of gVLM_B_ and SREM_B_ as complementary biomarkers helped to further minimize potential interindividual differences in ADME. A limitation regarding the use of biomarkers is that they better estimate intake at the time in which samples were collected. In this context, for a subset of participants we combined biomarkers levels estimated between 1- and 3-year follow-up, thus providing a wider time range for the biomarker-estimated flavanol intake assessment. Ultimately, biomarkers allow a better estimation of systemic exposure, which is key for the investigation of the health effect of dietary components. Similarly, the use of nutritional biomarkers facilitates the extrapolations of findings beyond the supplements tested in COSMOS. As shown in the case of biomarker-estimated assessment of the background diet, gVLM_B_ and SREM_B_ levels are also expected to increase after the consumption of flavanol-containing foods normally found in the diet, and thus, provide benchmarks for biomarkers levels that would be expected to mediate an effect.

We used nutritional biomarkers to segment participants into the biomarker-based active and control groups to explore the importance of background diet on the outcomes of RCTN. A limitation of this approach is that it results in differences in baseline characteristics of participants in biomarker active and biomarker control groups at baseline (Table S2) and breaks the randomization of the original trial. However, adjusting for these baseline differences (model 3, Table S3) shows that this has little impact on estimated outcomes and biomarker-based analyses still showed significant changes in HRs for all endpoints. Moreover, all were still larger than those determined via intervention-based ITT and PP analyses (Table 2).

COSMOS was used as a case study to explore the impact of background diet and adherence on RCTN outcomes. The findings from this work further confirm and strengthen the results reported in COSMOS main trial. Indeed, they suggest that there could be even stronger effects linked with flavanol intake than in the ITT and PP analysis in the COSMOS main trial (25). In addition, biomarker-estimated flavanol intake as part of background diet demonstrated that the amount of flavanols tested in COSMOS and similar to current recommendations (26) is achievable via a regular diet. In this context, further work will be needed to understand current flavanol intake levels in a US representative population. Such work will help assess the impact that increasing flavanol intake may have in the overall population.

### Conclusions

RCTN have an important role in the development of public health guidance, as they are considered the *gold standard* of evidence. In this study we have shown that the use of validated nutritional biomarkers is crucial to further strengthen the impact of RCTNs and increase scientific rigor and evidence quality as compared to RCT in medical research. Including biomarker-based assessments in RCTNs requires the need of rethinking current study designs commonly implemented in these studies, including i) the trade-off between number of volunteers recruited and possibility of collecting samples in the totality of the cohort for biomarker analysis, ii) the possibility of extending ITT and PP analyses to include biomarker-based endpoints as pre-registered outcomes in RCTNs, and iii) consider biomarker levels at baseline as part of inclusion/exclusion criteria (33). At the same time, more validated nutritional biomarkers are critically needed both for individual dietary compounds and dietary patterns for integration into RCTN design. This calls for further work aiming at the development and validation of nutritional biomarkers, which also includes investigating the ADME of dietary compounds under investigation and the analytical tools necessary to carry forward this research. There is a high cost in terms of funding, time and personnel needed to carry out RCTNs. Their success in testing hypotheses relies strongly on knowledge of background diet and accuracy in assessing adherence to the intervention. More funding to develop and validate nutritional biomarkers would enhance the scientific significance of RCTNs and subsequently their influence on diet and health recommendations and policy.

## Materials and Methods

### Study design

This study consisted of a post-hoc, secondary analysis of a subcohort (n=6,532) of COSMOS (NCT02422745; Supplemental Figure 1), a recently completed RCTN in 21,442 participants (including 8776 males ≥60 y and 12,666 females ≥65 y) in the US (25). The interventions included capsules containing cocoa extract that provided 500 mg/d cocoa flavanol or placebo, and a Centrum Silver daily multivitamin or placebo (provided by Pfizer Consumer Healthcare, now Haleon) in a 2×2 factorial design (see Supplementary Materials for details). Participants of the COSMOS Biomarker Cohort provided spot urine samples at baseline during the run-in phase of the study prior to randomization and again at 1, 2 and/or 3-year follow-up. Participants completed a self-reported adherence assessment every 6 months by answering a series of questions related to the number of days taking study pills (34). Enrolment period for the entire group of participants in COSMOS extended from April 2016 to March 2018, and the intervention was completed on December 31, 2020, with a median treatment period of 3.6 years. All participants provided written informed consent, and study approvals were obtained by the Institutional Review Board (IRB) at Mass General Brigham.

### Flavanol biomarker quantification

Urinary levels of 5-(3,4 -dihydroxyphenyl)-γ-valerolactone metabolites (gVLM_B_) and structurally related (−)-epicatechin metabolites (SREM_B_) were used as biomarkers. gVLM_B_ and SREM_B_ were validated as nutritional biomarkers of flavanol intake (23, 24). While gVLM_B_ informs on the intake of flavanols in general, SREM is a specific biomarker of the intake of (−)-epicatechin, one of the main bioactive flavanol compounds in the 500 mg/d CF tested in COSMOS. SREM_B_ and gVLM_B_ have different systemic half-lives (35), thus a combination of both biomarkers allows capturing different periods after flavanol intake. gVLM_B_ and SREM_B_ were quantified using validated LC-MS methods (36) and performance of the quantification are provided in Supplementary Information. Unadjusted biomarker concentrations were used as urinary creatinine is associated with CVD risk (27) and specific gravity did not materially change the outcome (see Supplementary Data for further details).

### Flavanol intake assessment using flavanol biomarkers

gVLM_B_ and SREM_B_ in spot urine are concentration biomarkers (21) and thus not suitable to calculate actual intake directly. We have therefore used a threshold approach to identify participants who consume at least 500 mg/d flavanols – the amount used in the COSMOS intervention – using data from the biomarker validation studies (23, 24). Thresholds are the lower 95% confidence intervals of the expected concentration of gVLM_B_ and SREM_B_ respectively, using a linear regression model (Supplemental materials and methods).

### Endpoints

Endpoints of this study were i) total cardiovascular disease (CVD) events, the primary outcome in the COSMOS main trial (myocardial infarction (MI), stroke, coronary revascularization, cardiovascular mortality, carotid artery surgery, peripheral artery surgery, and unstable angina requiring hospitalization); ii) CVD mortality, a secondary outcome in the COSMOS main trial; ii) all-cause mortality, a secondary outcome in the COSMOS main trial; and iv) major CVD events (MI, stroke, and CVD mortality), which are a recognized CVD outcomes, but was not included among pre-registered COSMOS outcomes. Participants reporting an outcome signed a release form to request related medical records for evaluation and processing according to standardized WHI and BWH study procedures. Self-reported primary and secondary outcomes were confirmed by medical record review by a committee of physicians and investigators blinded to treatment assignment. Further details on endpoint adjudication were published previously (25). Median follow-up time was 3.6 years.

### Statistical analysis

All analyses were conducted with R version 4.3.2 (37) in RStudio 2023.12.1 using the packages *rms* (38) for regression analyses and *ggplot2* (39) for the generation of graphics. Missing values were assumed to be missing at random and imputed using multiple imputation (including Nelson-Aalen estimator (40)). Unless indicated otherwise, results are shown with 95% confidence intervals (CI). Associations between endpoints and study groups were investigated using Cox proportional hazards models. The proportional hazard assumption was tested and confirmed with the *cox.zph* function. Statistical models were selected *a priori* based on analyses conducted previously (25). Continuous variables were transformed using restricted cubic splines with 3 knots (outer quantiles 0.1 and 0.9). Model 1 was adjusted by sex, age at randomization and recruitment cohort (Women’s Health Initiative or other). Model 2 was further adjusted by BMI (kg/m^2^), smoking status (never, ever, current), and aspirin use (yes/no). Model 3 was additionally adjusted by alternative Healthy Eating Index (aHEI, continuous), family history of hypertension, high cholesterol and stroke (categorical) and history of hypertension (categorical). Model 4 was like model 2, but additionally adjusted by randomization (Placebo or CF intervention), model 5 was like model 2, but additionally adjusted by aHEI. All results were shown as estimated hazard ratio (HR) (95% CI) between control and intervention. For per protocol analyses, participants were censored on the date when participants self-reported missing either more than 8 pills per month or that they were unsure how many pills they have missed, or began outside non-study use of cocoa supplement. Cumulative incidence plots were created using the *survplot* function and data from model 2 (including age at randomization, sex, BMI, smoking status, aspirin use and recruitment cohort).

## Supplementary Material

Supplement 1

## Figures and Tables

**Table 1.** Number of participants with flavanol biomarker levels consistent with a flavanol intake of 500 mg/d or higher at baseline and follow-up.

	Biomarker-estimated flavanol intake ≥ 500 mg/d	
	Baseline (background diet)	Follow-up (adherence)
Participants with spot urine at baseline (n=6,509)	1,249 (19%)	—
Placebo (n=3,257)	603 (19%)	—
CF intervention (n=3,252)	646 (20%)	—
Participants with spot urine at baseline and follow-up (n=2,051)	414 (20%)	881 (43%)
Placebo (n=991)	186 (19%)	175 (18%)
CF intervention (n=1,060)	228 (22%)	706 (67%)

**Table 2.** HRs and 95% CIs for cardiovascular (CVD) outcomes, according to randomized assignment in intention-to-treat (ITT) and per-protocol (PP) analyses and according to biomarker-based groups. Summary statistics were from Cox regression models adjusted by age, sex, BMI, smoking status, recruitment cohort and aspirin use (model 2). CIs were not adjusted for multiple comparisons.

	Intervention-based groups		Biomarker-based groups
	ITT^†^ (n=6,532)	PP^‡^ (n=6,532)	Biomarker (n=4,735)
All-cause mortality	0.81 (0.61; 1.08)	0.69 (0.45; 1.05)	0.54 (0.37; 0.80)
Total CVD events	0.83 (0.65; 1.07)	0.79 (0.59; 1.05)	0.65 (0.47; 0.89)
CVD mortality	0.53 (0.29; 0.96)	0.51 (0.23; 1.14)	0.44 (0.20; 0.97)
Major CVD events	0.75 (0.55; 1.02)	0.62 (0.43; 0.91)	0.48 (0.31; 0.74)

Total CVD events is a composite of myocardial infarction, stroke, CVD death, coronary artery bypass graft and percutaneous coronary intervention, unstable angina including hospitalization, carotid artery surgery, and peripheral artery surgery. Major CVD events was a composite of myocardial infarction, stroke, and CVD death. CVD, cardiovascular disease;

† Intention to treat;

‡ per-protocol

## Data Availability

Code is available from https://gitlab.act.reading.ac.uk/xb901875/biomarker-base-dintervention. Data and associated documentation will be available to users only under a data-sharing agreement. Details on the availability of the study data to other investigators will be on our study website at https://cosmostrial.org/.
