# Supplementary material for: The overlooked impact of background diet and adherence in nutrition trials
Source: medRxiv. 2025 Feb 26:2025.02.26.25322933. Preprint. [Version 1] doi: 10.1101/2025.02.26.25322933 (PMC11888514; doi:10.1101/2025.02.26.25322933)
Supplement: Supplementary file 1 [file media-1.pdf]

Supporting Information for

Transforming nutrition research: the overlooked impact of background diet and adherence.

Javier I. Ottaviani[1], Hagen Schroeter[1], Dennis M. Bier[2], John W. Erdman Jr[3], Howard D. Sesso[4], JoAnn E. Manson[4], Gunter G. C. Kuhnle*[5]

Corresponding author: Gunter G. C. Kuhnle

Email: g.g.kuhnle@reading.ac.uk

Supporting Information Text

Supplemental materials and methods

Study design

This study consisted of a post-hoc, secondary analysis of data emanated from a subcohort (n=6,532) of the COcoa Supplement and Multivitamin Outcomes Study (COSMOS, NCT 02422745), which is a recently completed randomized clinical trial in nutrition (RCTN) in 21,442 participants (including 8776 males ≥60 y and 12,666 females ≥65 y) in the US (1, 2). COSMOS followed a 2×2 factorial, randomized, double-blind, placebo-controlled design, and aimed at assessing the effects of cocoa extract (mainly containing cocoa flavanols) and a multivitamin supplement on total cardiovascular disease and total invasive cancer, respectively. The interventions included capsules containing cocoa extract that provided 500 mg/d cocoa flavanol (including 80 mg (–)-epicatechin) or a placebo (provided by Mars Edge) and a Centrum Silver daily multivitamin or placebo (provided by Pfizer Consumer Healthcare, now Haleon). For this study, we report the effects of the cocoa extract intervention.

The trial included women aged ≥65 y and men aged ≥60 y. Additional inclusion criteria were willingness to participate in the trial as evidenced by completion of informed consent and baseline forms. Major exclusion criteria were history of myocardial infarction or stroke; diagnosis of invasive cancer other than nonmelanoma skin cancer in the last 2 y prior to enrollment; serious illness that would preclude participation; taking cocoa extract, multivitamins, high-dose vitamin D, or high-dose calcium supplements and not willing to forego use during the trial; extreme sensitivity to caffeine; less than 75% compliance to study procedures during at least a 2-mo run-in period; and inability to communicate in English. COSMOS recruited participants from three main sources, including the Women's Health Imitative (WHI), respondents to recruitment efforts for the VITamin D and OmegA-3 TriaL (NCT01169259) who were not ultimately randomized into the trial, and those identified through mass mailings and media efforts.

The population investigated in this study included a subcohort of 6,532 participants who provided spot urine samples during their participation in COSMOS [hereafter referred as COSMOS Biomarker cohort] (**Figure S1**). Urine samples were provided at baseline during the run-in phase of the study and at 1, 2 and/or 3-year follow-up. During the trial, participants completed a self-reported adherence assessment every 6 month by answering a series of questions related to the number of days taking study pills.

Enrollment period for the entire group of participants in COSMOS extended from April 2016 to March 2018, and the intervention was completed on December 31, 2020, with a median treatment period of 3.6 y. Further details of the protocol and main findings of the study were previously published [ref]. All participants provided written informed consent, and study approvals were obtained by the Institutional Review Board (IRB) at Mass General Brigham.

Flavanol biomarker quantification

Two flavanol biomarkers were assessed, including 5-(3',4'-dihydroxyphenyl)-γ-valerolactone metabolites ($gVLM_B$) and structurally related (–)-epicatechin metabolites ($SREM_B$). $gVLM_B$ represents the sum of two specific phase II metabolites of gut microbiota-derived flavanol catabolites, including 5-(4′-hydroxyphenyl)-γ-valerolactone-3′-sulfate and 5-(4′-hydroxyphenyl)-γ-valerolactone-3′-glucuronide. $SREM_B$ represents the sum of three phase II flavanol metabolites, including (–)-epicatechin-3′-glucuronide, (–)-epicatechin-3′-sulfate and 3′-methyl-(–)-epicatechin-5-sulfate. Both, $gVLM_B$ and $SREM_B$ were previously validated as nutritional biomarkers and proved to provide

complementary information about flavanol intake (3, 4). While $gVLM_B$ informs on the intake of flavanols in general, SREM is a specific biomarker of the intake of (–)-epicatechin, one of the main bioactive flavanol present within the 500 mg/d CF tested in COSMOS (5). In addition, with $gVLM_B$ having a longer systemic half-life than SREM (6), the combination of both biomarkers allows capturing different periods after flavanol intake and thus, increase the chance of identifying flavanol consumption in volunteers.

$gVLM_B$ and $SREM_B$ were quantified using validated methods based on UPLC-MS with authentic standards and corresponding $^{13}C/^{2}H$-labeled internal standards (3, 4). Quality assurance was performed as described previously (7): each batch included two replicates of three performance quality control (QC) and one monitoring QC sample with known concentrations of biomarker. The acceptance criteria for each batch were that at least one performance QC at each concentration and four out of the six performance QCs were within 15% of the theoretical concentration. Concentrations outside the limit of quantifications were imputed by the values reported. This provides a better estimate of actual concentration than imputation by a fixed valued.

Flavanol biomarkers were measured in urine samples collected at baseline (n=6509), year 1 (n=1830), year 2 (n=1918) and year 3 (n=1050). Those volunteers who collected more than one urine sample at follow-up, corresponding concentrations of $gVLM_B$ and $SREM_B$ were averaged to simplify to one follow-up flavanol biomarker concentration per volunteer (n=2,051).

Adjustment of spot urine samples

Spot urine samples are often adjusted using urinary creatinine, although this method is likely to introduce bias (8). Specific gravity has been suggested as an alternative (9) and has been used previously (7), although it is not clear whether such an adjustment is always required. In this study we have therefore investigated a subset of urine samples (n=490) and determined specific gravity (1.02 (95% CI 1.01; 1.02); median (IQR)). Adjusting $gVLM_B$ and $SREM_B$ concentrations by specific gravity resulted in a median (IQR) concentration change of 0.05 (95% CI 0.01; 0.18) µmol/L or relative change of 1.5 (1.1; 2.0)%, but did not significantly alter ranking of participants (see Bland-Altman plot (10) in **Figure S2**). Thus, we determined that adjusting flavanol biomarkers levels not necessary for this study.

Flavanol intake assessment using flavanol biomarkers

The quantification of gVLM and SREM in spot urine serve as concentration biomarkers of flavanol intake and thus, these biomarkers could not be used to determine absolute flavanol consumption (11). Instead, gVLM and SREM concentrations were used to classify participants into having flavanol intakes either below or equal and above the 500 mg/d tested in COSMOS. Thresholds for gVLM and SREM concentrations were derived from a dose-escalation study conducted as part of the validation of these flavanol biomarkers (3, 4). Considering the inter-individual variability in biomarker levels and to reduce the likelihood of false negatives, respective thresholds were defined as the bottom 95% CI limit of the expected concentration of gVLM and SREM after the intake of 500 mg of CF (**Figure S3**) using a linear regression model with log_2-transformed concentration data. These values were determined as 18.2 µM for gVLM and 7.8 µM for SREM.

Sensitivity analysis of threshold

Given the importance of thresholds selected to define the biomarker-based CF active and control groups, we investigated the impact of using different thresholds for $gVLM_B$ and $SREM_B$ to identify participant with flavanol intake of at least 500 mg/d. We have estimated associations between biomarker status (above threshold for $gVLM_B$ **or** $SREM_B$

[biomarker active] vs. below threshold for $gVLM_B$ **and** $SREM_B$ [biomarker control]) for thresholds varying between 1 μM and 50 μM for $gVLM_B$ and 1 μM and 30 μM for $SREM_B$. The results show that thresholds for $gVLM_B$ varying between 3 to 20 μM and thresholds for $SREM_B$ varying between 5 to 30 μM would have also resulted in significant risk reductions for total CVD events, CVD deaths and all-cause deaths between biomarker active and control groups (**Figure S4**). In the case of major CVD events, significant differences between groups were observed when gVLM threshold varied between 3 to 20 μM and $SREM_B$ threshold varied between 3 to 12 μM. Thus, the thresholds of 18.2 μM for $gVLM_B$ and 7.8 μM for SREM determined for this study fall well within these ranges, and even considering potential inaccuracies in the approach chosen to determine the thresholds used in this study would have not changed the outcomes of this analysis.

Supplemental Figures

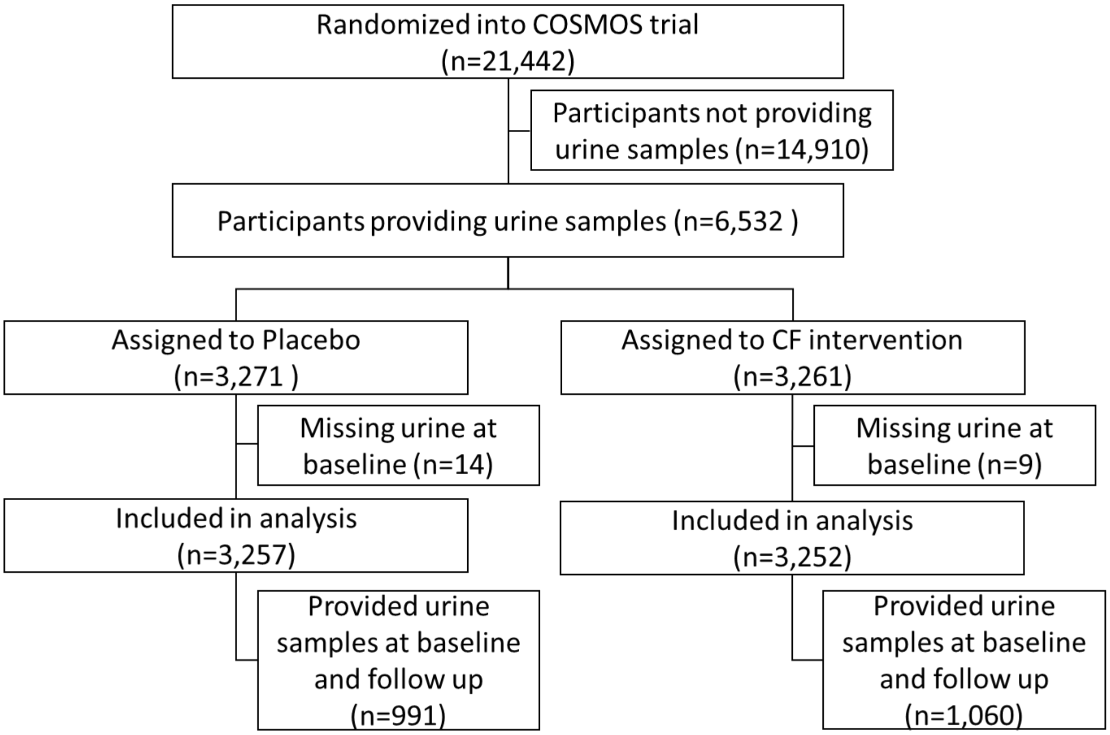

Figure S1. CONSORT Diagram of the COSMOS Biomarker Cohort

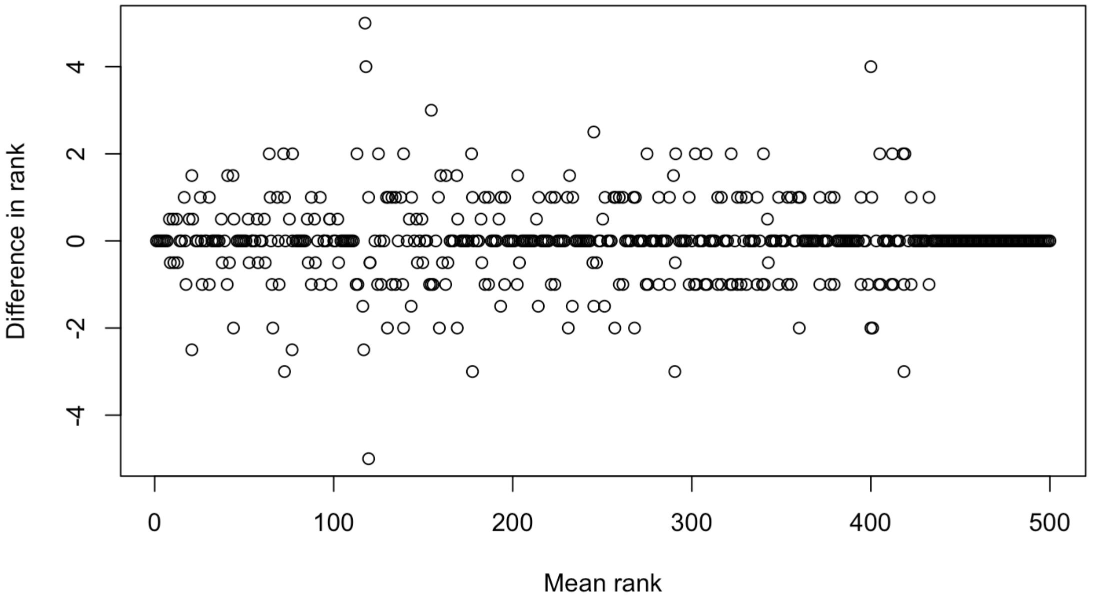

Figure S2: Bland-Altman plot (10) comparing participant rank of biomarker with and without adjustment for specific gravity. Difference in ranking of participants by flavanol biomarker concentrations in urine before and after adjusting by specific gravity.

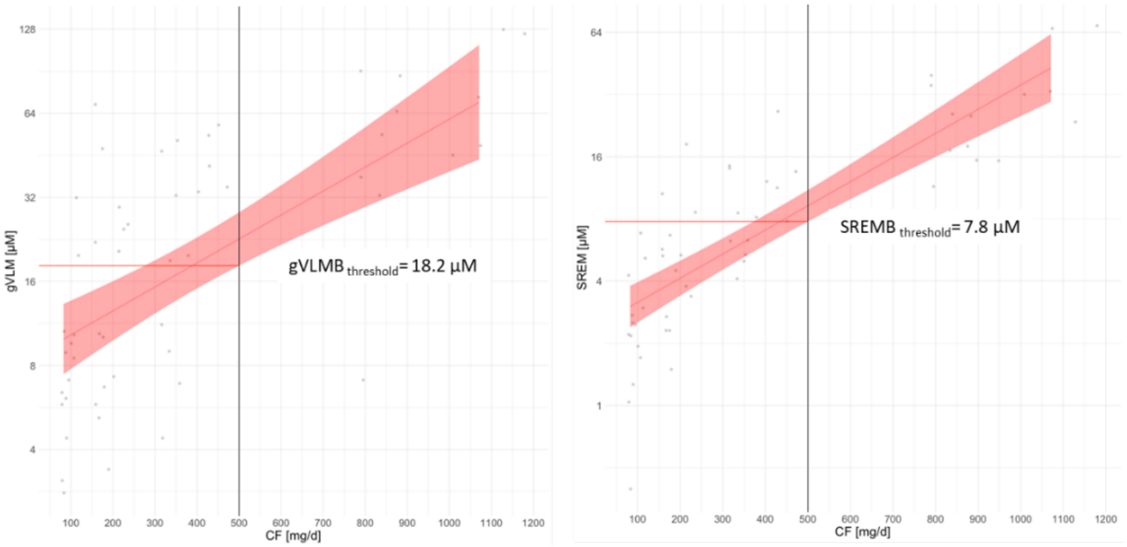

Figure S3: Concentration of gVLM (A) and SREM (B) as a function of the amount of cocoa flavanols consumed. Flavanol biomarker data was log-transformed. gVLM and SREM thresholds were defined as the bottom 95%CI limit of the expected concentration of gVLM and SREM after the intake of 500 mg of cocoa flavanols. Data were obtained from data collected previously (3, 4).

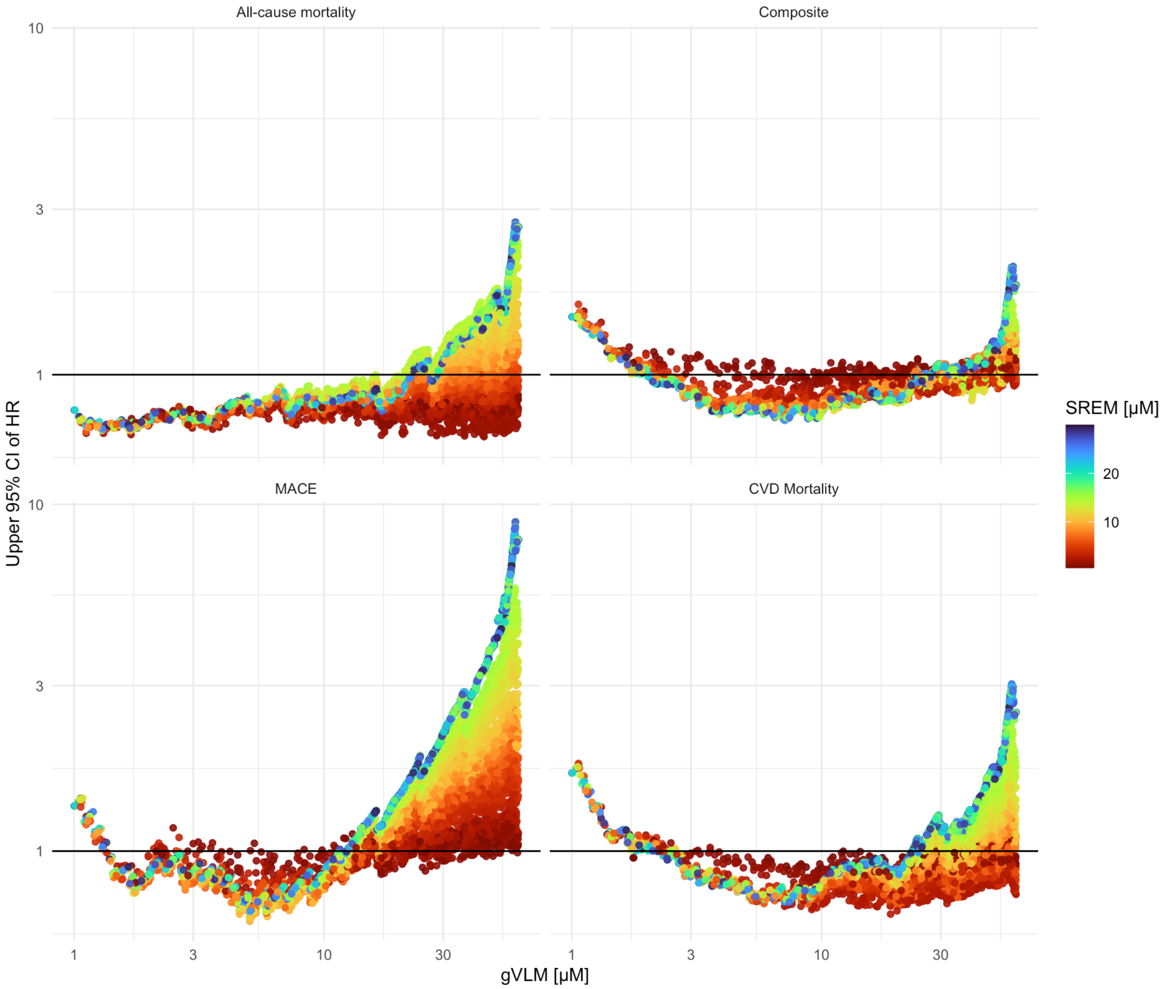

Figure S4: Comparison of 95% upper confidence limit of HR for different CVD events between biomarker-active and biomarker-control group when using different concentration thresholds. Summary statistics were from Cox regression models by age, sex, BMI, smoking status, and aspirin use. CIs were not adjusted for multiple comparisons. CVD events included total CVD events (which was a composite of myocardial infarction, stroke, CVD death, coronary artery bypass graft and percutaneous coronary intervention, unstable angina including hospitalization, carotid artery surgery, and peripheral artery surgery), CVD deaths, all-cause deaths and major adverse CVD events (which was a composite of myocardial infarction, stroke, and CVD death).

Supplemental Tables

Table S1. Characteristics of the study population. Data are mean ± SD or proportion. Test for difference between participants with and without urine samples using t-test or χ^2 test.

	Total COSMOS Cohort	Participant who did not provide urine samples	Participant who provided urine samples [COSMOS-Biomarker cohort]	p
n	21,442	14,910	6,532	
Cocoa-flavanol intervention	10,719 (50%)	7458 (50%)	3261 (50%)	0.908
n of women	12666 (59%)	9325 (63%)	3341 (51%)	<0.001
Age (y)	72.1 ± 6.6	72.6 ± 6.6	71.0 ± 6.3	<0.001
BMI (kg/m2)	27.7 ± 5.4	27.7 ± 5.4	27.6 ± 5.3	0.094
Smoking status				0.317
Never (n)	11,565 (55%)	8066 (55%)	3499 (54%)	
Ever (n)	8731 (41%)	6018 (41%)	2713 (42%)	
Current (n)	835 (4%)	591 (4 %)	244 (4%)	
Participants from WHI cohort (n)	4611 (22%)	3639 (24%)	972 (15%)	<0.001
Aspirin use (n)	10379 (49%)	7182 (49%)	3197 (49%)	0.388
Cholesterol medication use (n)	9405 (44%)	6480 (44%)	2925 (45%)	0.115
History of CVD (n)	1269 (6%)	882 (5.9)	387 (5.9)	1.000
History of hypertension (n)	12423 (58%)	8752 (59%)	3671 (56%)	0.001
CVD Risk factors (n)				0.914
0-1	9159 (43%)	6376 (43%)	2783 (43%)	
2	6289 (29%)	4358 (29%)	1931 (30%)	
≧3	5901 (28%)	4101 (28%)	1800 (28%)	
Diet quality (aHEI†, %)	42.4 ± 11.0	42.3 ± 11.0	42.6 ± 10.8	0.182
**CVD Events**				
Total CVD events (n)	866 (4%)	609 (4%)	257 (4%)	0.634
CVD mortality (n)	180 (1%)	131 (1%)	49 (1%)	0.386
All-cause mortality (n)	750 (3.5)	556 (4%)	194 (3%)	0.006
Major cardiovascular events (n)	559 (2.6)	396 (3%)	163 (3%)	0.527
**Biomarker concentration (median [IQR])**				
gVLM at baseline (µmol/L)	—	—	3.3 [0.8, 11.0]	
gVLM at follow-up (µmol/L)	—	—	8.0 [2.3, 23.2]	
SREM at baseline (µmol/L)	—	—	0.6 [0.3, 2.0]	
SREM at follow-up (µmol/L)	—	—	2.9 [0.8, 8.1]	

†alternative Healthy Eating Index

Table S2. Participant characteristics of treatment groups and biomarker-based active and control groups. Data shown are mean ± SD or count and proportion. P-value is for test for difference between randomised groups and between biomarker-based groups using t-test or χ^2 test.

	Randomised/Treatment assigned			Biomarker-based		
	Placebo	Cocoa flavanols	p	Biomarker control	Biomarker active	p
n	3271	3261		2,841	1,894	
n of participants randomized to CF pills (%)	0 (0.0)	3261 (100%)	**<0.001**	298 (11%)	1180 (62%)	**<0.001**
n of women (%)	1679 (51%)	1662 (51%)	0.788	1529 (54%)	841 (44%)	**<0.001**
Age (y)	71.0± 6.3	71.0 ± 6.3	0.94	71.3 ± 6.3	70.3 ± 6.2	**<0.001**
BMI (kg/m2)	27.7 ± 5.3	27.5± 5.3	0.129	27.7 ± 5.4	27.4 ± 5.0	**0.02**
Smoking status			0.486			0.516
Never (n)	1741 (54%)	1758 (55%)		1519 (54%)	1022 (55%)	
Ever (n)	1363 (42%)	1350 (42%)		1176 (42%)	788 (42%)	
Current (n)	131 (4%)	113 (4%)		116 (4%)	65 (4%)	
Participants from WHI cohort	504 (15%)	468 (14%)	0.244	454 (16%)	220 (12%)	**<0.001**
Aspirin use (n)	1592 (49%)	1605 (50%)	0.701	1384 (49%)	942 (50%)	0.579
Cholesterol medication use (n)	1448 (45%)	1477 (45%)	0.528	1271 (45%)	828 (44%)	0.413
History of CVD (n)	207 (6%)	180 (6%)	0.183	184 (7%)	100 (5%)	0.102
History of hypertension (n)	1850 (57%)	1821 (56%)	0.529	1637 (58%)	1016 (54%)	**0.006**
CVD risks			0.227			**0.004**
0-1 (n)	1382 (42%)	1401 (43%)		**1181 (47%)**	**852 (45%)**	
2 (n)	947 (29%)	984 (30%)		**820 (29%)**	**570 (30%)**	
≧3 (n)	931 (29%)	869 (27%)		**828 (29%)**	**472 (25%)**	
Diet quality (aHEI†, %)	42.7 ± 11.0	42.5 ± 10.7	0.538	42.4 ± 10.9	43.3 ± 10.8	**0.007**
CVD events						
Total CVD events (n)	141 (4%)	116 (4%)	0.133	128 (5%)	56 (3%)	**0.009**
CVD mortality (n)	32 (1%)	17 (0.5%)	**0.046**	29 (1%)	8 (0%)	**0.034**
All-cause mortality (n)	108 (3%)	86 (3%)	0.131	100 (4%)	34 (2%)	**0.001**
Major CVD events (n)	94 (3%)	69 (2%)	0.050	89 (3%)	28 (2%)	**<0.001**
Biomarker concentration (median [IQR])						
gVLM baseline (µmol/L)	3.4 [0.8, 11.1]	3.21 [0.8, 10.9]	0.49	2.0 [0.5, 5.7]	20.9 [4.6, 36.2]	**<0.001**
gVLM follow-up (µmol/L)	4.2 [1.4, 10.9]	16.04 [5.1, 34.9]	**<0.001**	3.2 [1.0, 7.3]	22.5 [8.5, 37.7]	**<0.001**
SREM baseline (µmol/L)	0.6[0.3, 1.8]	0.61 [0.3, 2.1]	0.187	0.4 [0.3, 1.2]	1.5 [0.4, 5.3]	**<0.001**
SREM follow-up (µmol/L)	1.0 [0.4, 2.4]	7.41 [3.3, 11.5]	**<0.001**	1.1 [0.4, 2.8]	7.9 [2.6, 11.6]	**<0.001**

†alternative Healthy Eating Index

Table S3: Sensitivity analyses. HRs and 95% CIs for CVD outcomes using biomarker-based groups using different models. Summary statistics from Cox regression models show HRs comparing *biomarker control* and *biomarker active* group for each outcome. CIs were not adjusted for multiple comparisons.

	Model 1†	Model 2†	Model 3†	Model 4†	Model 5†
All-cause mortality	0.52 (0.35; 0.77)	0.54 (0.37; 0.80)	0.57 (0.39; 0.85)	0.65 (0.41; 1.02)	0.55 (0.37; 0.82)
Total CVD events	0.64 (0.47; 0.88)	0.65 (0.47; 0.89)	0.68 (0.49; 0.93)	0.70 (0.48; 1.02)	0.66 (0.48; 0.90)
CVD mortality	0.42 (0.19; 0.93)	0.44 (0.20; 0.97)	0.46 (0.21; 1.02)	0.64 (0.26; 1.54)	0.45 (0.20; 0.98)
Major CVD events	0.48 (0.31; 0.73)	0.48 (0.31; 0.74)	0.50 (0.33; 0.78)	0.55 (0.33; 0.91)	0.49 (0.32; 0.75)

Total CVD events is a composite of myocardial infarction, stroke, CVD death, coronary artery bypass graft and percutaneous coronary intervention, unstable angina including hospitalization, carotid artery surgery, and peripheral artery surgery. Major CVD events was a composite of myocardial infarction, stroke, and CVD death. CVD, cardiovascular disease; †Model 1 was adjusted by sex, age at randomization and recruitment cohort (Women's Health Initiative or other). Model 2 was further adjusted by BMI (kg/m^2), smoking status (never, ever, current), and aspirin use (yes/no). Model 3 was additionally adjusted by alternative Healthy Eating Index (aHEI, continuous), family history of hypertension, high cholesterol and stroke (categorical) and history of hypertension (categorical). Model 4 was like model 2, but additionally adjusted by randomization (Placebo or CF intervention), model 5 was like model 2, but additionally adjusted by aHEI.

SI References

1. H. D. Sesso *et al.*, Effect of cocoa flavanol supplementation for the prevention of cardiovascular disease events: the COcoa Supplement and Multivitamin Outcomes Study (COSMOS) randomized clinical trial. *Am J Clin Nutr* **115**, 1490-1500 (2022).
2. P. M. Rist *et al.*, Design and baseline characteristics of participants in the COcoa Supplement and Multivitamin Outcomes Study (COSMOS). *Contemp Clin Trials* **116**, 106728 (2022).
3. J. I. Ottaviani *et al.*, Evaluation of (−)-epicatechin metabolites as recovery biomarker of dietary flavan-3-ol intake. *Scientific reports* **9**, 1-10 (2019).
4. J. I. Ottaviani *et al.*, Evaluation at scale of microbiome-derived metabolites as biomarker of flavan-3-ol intake in epidemiological studies. *Scientific Reports* **8**, 1-11 (2018).
5. J. I. Ottaviani *et al.*, The stereochemical configuration of flavanols influences the level and metabolism of flavanols in humans and their biological activity in vivo. *Free Radical Biology and Medicine* **50**, 237-244 (2011).
6. J. I. Ottaviani *et al.*, The metabolome of [2-14C](−)-epicatechin in humans: implications for the assessment of efficacy, safety, and mechanisms of action of polyphenolic bioactives. *Scientific reports* **6**, 1-10 (2016).
7. J. I. Ottaviani *et al.*, Biomarker-estimated flavan-3-ol intake is associated with lower blood pressure in cross-sectional analysis in EPIC Norfolk. *Scientific reports* **10**, 17964 (2020).
8. J. I. Ottaviani, H. Schroeter, G. G. C. Kuhnle, Measuring the intake of dietary bioactives: Pitfalls and how to avoid them. *Mol Aspects Med* **89**, 101139 (2023).
9. S. A. Bingham *et al.*, Epidemiologic assessment of sugars consumption using biomarkers: comparisons of obese and nonobese individuals in the European prospective investigation of cancer Norfolk. *Cancer epidemiology, biomarkers & prevention : a publication of the American Association for Cancer Research, cosponsored by the American Society of Preventive Oncology* **16**, 1651-1654 (2007).
10. J. Bland, D. G. Altman, Statistical methods for assessing agreement between two methods of clinical measurement. *Lancet* **327**, 307-310 (1986).
11. R. Kaaks, E. Riboli, R. Sinha, Biochemical markers of dietary intake. *IARC Scientific Publication No. 84*, 103-126 (1997).
